# The ideal intravitreal injection setting: office, ambulatory surgery room or operating theatre? A narrative review and international survey

**DOI:** 10.1007/s00417-023-06108-y

**Published:** 2023-05-18

**Authors:** Daniele Veritti, Valentina Sarao, Jay Chhablani, Anat Loewenstein, Paolo Lanzetta, Società Italiana della Retina (SIR) Study Group, Francesco Bandello, Edoardo Midena, Massimo Nicolò, Mariacristina Parravano, Elisabetta Pilotto, Federico Ricci, Giovanni Staurenghi, Gianni Virgili, Intravitreal Injection Setting Study Group (I2SG), Jennifer J. Arnold, Albert J. Augustin, Catherine Creuzot-Garcher, Monica Lövestam Adrian, Polona Jaki Mekjavić, Praveen J. Patel, Francisco J. Rodriguez, Ricarda Schumann, Ashish Sharma, Rufino Silva, Javier Zarranz-Ventura

**Affiliations:** 1 Department of Medicine - Ophthalmology, University of Udine, Piazzale Santa Maria Della Misericordia, 33100 Udine, Italy; 2 Istituto Europeo Di Microchirurgia Oculare - IEMO, Udine, Italy; 3 Department of Ophthalmology, UPMC Eye Center, University of Pittsburgh, Pittsburgh, PA USA; 4 Division of Ophthalmology, Tel Aviv Medical, Tel Aviv University, Tel Aviv, Israel

**Keywords:** Ambulatory surgery room, Endophthalmitis, In-office, Intravitreal injection, Operating theatre, Setting

## Abstract

**Purpose:**

This study reviews evidence and provides recommendations for the ideal setting of intravitreal injection (IVI) administration of vascular endothelial growth factor (VEGF) inhibitors.

**Methods:**

A multi-step approach was employed, including content analysis of regulations and guidelines, a systematic literature review, and an international survey assessing perioperative complications and endophthalmitis incidence in relation to injection settings. The literature review searched PubMed and Cochrane databases from 2006 to 2022, focusing on studies reporting correlations between complications and treatment settings. The survey utilized a web-based questionnaire distributed to clinical sites and the international ophthalmic community, with data managed using electronic capture tools.

**Results:**

We reviewed regulations and guidelines from 23 countries across five continents, finding significant variation in IVI administration settings. In most countries, IVI is primarily administered in outpatient clean rooms (96%) or offices (39%), while in others, it is restricted to ambulatory surgery rooms or hospital-based operating theatres (4%). The literature review found that endophthalmitis risk after IVI is generally low (0.01% to 0.26% per procedure), with no significant difference between office-based and operating room settings. The international survey (20 centers, 96,624 anti-VEGF injections) found low overall incidences of severe perioperative systemic adverse events and endophthalmitis, independent of injection settings.

**Conclusion:**

No significant differences in perioperative complications were observed among various settings, including operating theatres, ambulatory surgery rooms, offices, hospitals, or extra-hospital environments. Choosing the appropriate clinical setting can optimize patient management, potentially increasing effectiveness, quality, productivity, and capacity.

**Supplementary Information:**

The online version contains supplementary material available at 10.1007/s00417-023-06108-y.


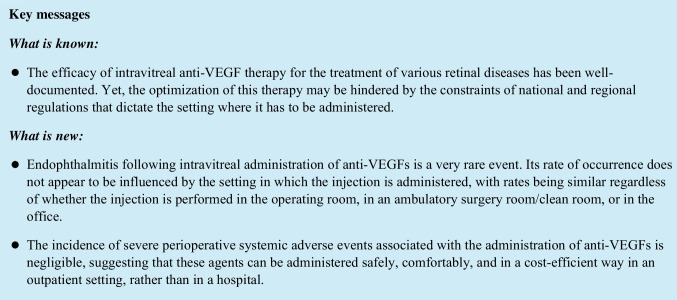


## Introduction

Intravitreal injection (IVI) of vascular endothelial growth factor (VEGF) inhibitors is the gold standard treatment for various retinal and choroidal diseases [1–7]. Intravitreal delivery allows adequate intraocular concentration, minimizing systemic exposure [8]. These inhibitors have the potential to reduce blindness and visual impairment [9]. However, to fully optimize the impact of intravitreal anti-VEGF therapies on global health and blindness prevalence, obstacles to its widespread utilization should be analyzed and possibly removed. One of the key limitations is the setting in which intravitreal anti-VEGF therapy is given, following national and regional restrictions. There is a significant variation between countries in terms of regulations and guidelines. For example, in Italy, intravitreal anti-VEGF agents can only be administered in highly specialized hospitals that have been authorized by each regional government [10]. In contrast, in many other countries, intravitreal agents can be given in an ambulatory located outside the hospital, or even in medical offices [11, 12]. Although IVI is relatively safe, severe ocular adverse events, including endophthalmitis, may occur and its occurrence may be related to procedural and environmental factors [13, 14]. Perioperative systemic safety might also differ depending on the setting. The possibility of delayed emergency care in office outpatient settings must be balanced against overall safety of the treatment, which may not actually increase the incidence of perioperative systemic adverse events. Despite numerous studies on postoperative safety of intravitreal anti-VEGF inhibition, systemic complications during treatment at medical facilities have received relatively scarce attention.

This work aims to review the medical literature and provide evidence-based recommendations using a multi-step approach:

1. Overview of country-specific regulations and guidelines regarding intravitreal anti-VEGF administration settings;
2. Literature review of setting-related ocular and systemic complications;
3. International survey to quantify perioperative complications and endophthalmitis incidence in relation to intravitreal therapy settings.

## Methods

### Regulations and guidelines

We analyzed policy documents, regulations, and guidelines on intravitreal anti-VEGF treatment from electronic databases, references, and government repositories. The study questions were: in what setting is intravitreal anti-VEGF administration permitted in a specific country?; Is intravitreal anti-VEGF treatment allowed outside of a hospital setting in a specific country?. Country selection was based on relevance, literature contribution, and document availability. Electronic databases such as PubMed, Embase, PsychINFO, Google Scholar, and the National Guideline Clearinghouse were searched. A targeted Google search was also conducted, considering the first 100 relevant results per country. Two investigators (DV and VS) independently reviewed the documents and reached a consensus. Supplementary Table 1 details definitions of different intravitreal settings.

### Literature review

A systematic search of PubMed and Cochrane databases was conducted to analyze the safety profiles of intravitreal anti-VEGF therapy from 2006–2022. Information from clinical trials, meta-analyses, reviews, observational studies, and case reports was gathered. Key terms included “age-related macular degeneration”, “choroidal neovascularization”, “anti-vascular endothelial growth factor therapy”, “bevacizumab”, “ranibizumab”, “aflibercept”, “systemic adverse events”, “ocular adverse events”, and “intravitreal injection”. Secondary searches involved cited articles in reference lists. Only English studies were included. Studies reporting on perioperative complications and endophthalmitis in relation to the setting in which intravitreal therapy was administered were considered of particular interest. Severe adverse events were defined as reactions resulting in death, life-threatening situations, hospitalization, disability, or requiring medical intervention. A perioperative severe adverse event was defined as a severe adverse event occurring during the patient’s stay for anti-VEGF IVI.

### Survey

An internet-based survey was designed to collect information on procedural settings, severe perioperative systemic adverse events, and ocular complications linked to intravitreal treatment. A participation request was sent to renowned experts in intravitreal therapies (Intravitreal Injection Setting Study Group—I2SG) and disseminated via email to the international ophthalmic community. The questionnaire requested data on anti-VEGF injections in 2019, specific adverse events, and post-injection endophthalmitis (Supplementary Table 2). Data were collected and managed using electronic data capture tools, featuring a secure web-based application with validated data entry, audit trails, and automated export procedures.

## Results

### Regulations and guidelines

**Fig. 1:**
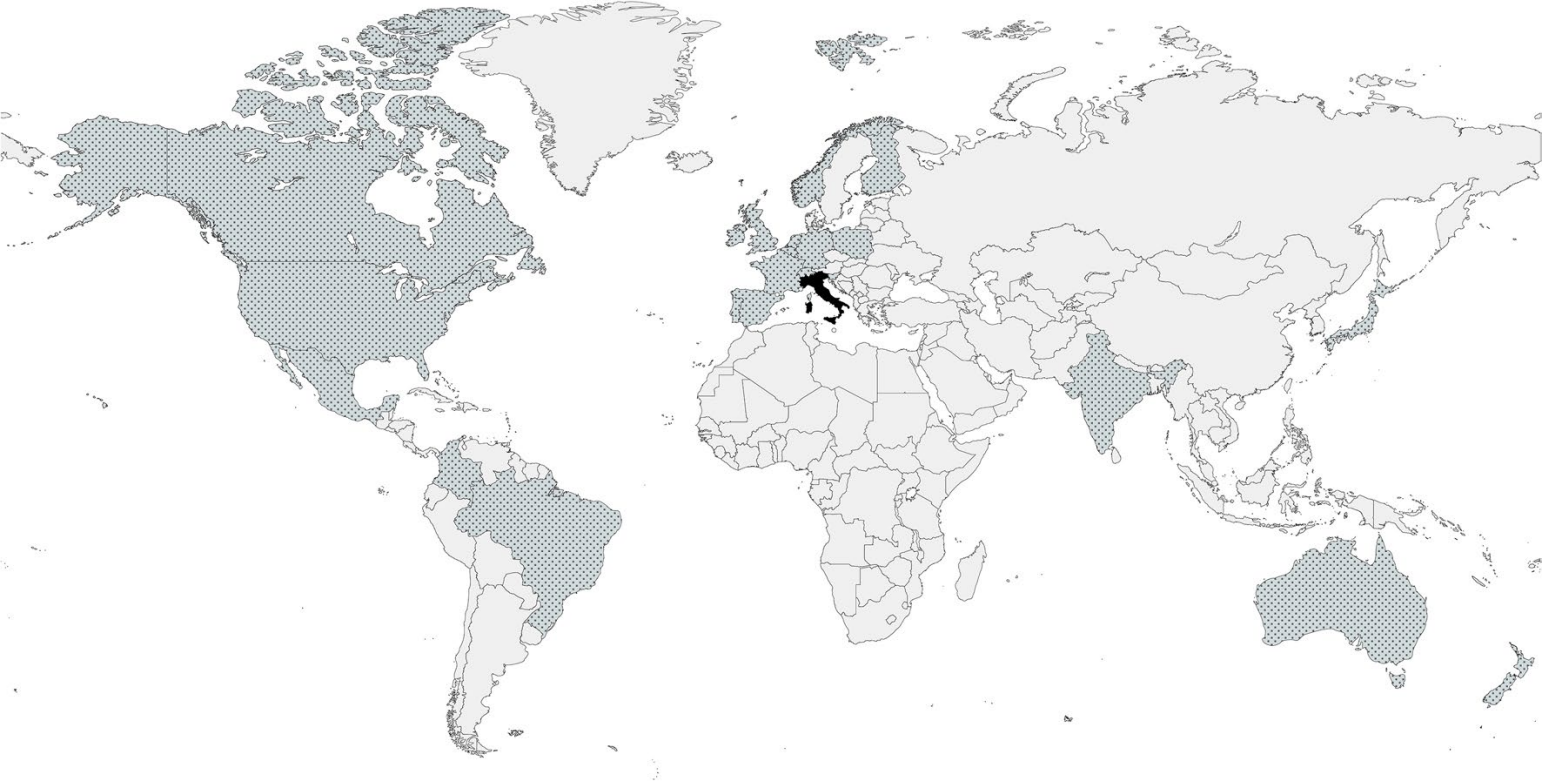
Countries that allow intravitreal anti-VEGF administration either outside the hospital (dotted) or exclusively within hospital settings or analogous healthcare facilities (black)

Our comprehensive search for guidelines on IVI administration settings yielded results from 23 countries across five continents (Table 1, Fig. 1) [10, 15–35]. All 23 countries allow IVIs of anti-VEGFs in ambulatory surgery rooms/clean rooms, and in 9 (39%), medical offices are permitted. All countries, except Italy, allow intravitreal anti-VEGF use outside hospitals.

**Table 1:** Country-specific regulations and guidelines regarding the settings where intravitreal anti-VEGFs can be administered

Country	Continent	Intravitreal setting			Intravitreal anti-VEGF injection allowed outside hospital?
		Operating theatre	Ambulatory surgery room/Clean room	Office	
Australia	Australia/Oceania	YES	YES	YES	YES
Belgium	Europe	YES	YES	NO	YES
Brazil	South America	YES	YES	YES	YES
Canada	North America	YES	YES	YES	YES
Colombia	South America	YES	YES	NO	YES
Finland	Europe	YES	YES	NO	YES
France	Europe	YES	YES	YES	YES
Germany	Europe	YES	YES	NO	YES
India	Asia	YES	YES	YES	YES
Ireland	Europe	YES	YES	NO	YES
Italy	Europe	YES	YES	NO	NO (*)
Japan	Asia	YES	YES	YES	YES
Mexico	North America	YES	YES	YES	YES
Netherlands	Europe	YES	YES	NO	YES
New Zealand	Australia/Oceania	YES	YES	YES	YES
Norway	Europe	YES	YES	NO	YES
Poland	Europe	YES	YES	NO	YES
Portugal	Europe	YES	YES	NO	YES
Slovenia	Europe	YES	YES	NO	YES
Spain	Europe	YES	YES	NO	YES
Switzerland	Europe	YES	YES	NO	YES
United Kingdom	Europe	YES	YES	NO	YES
United States	North America	YES	YES	YES	YES

Legend: (*) Intravitreal anti-VEGFs are allowed only in hospital settings (or analogous healthcare facilities); VEGF: vascular endothelial growth factor

### Literature review

#### Endophthalmitis

Endophthalmitis, a severe eye inflammation, can result in irreversible blindness if left untreated [36]. Fortunately, the risk of endophthalmitis following IVIs is generally extremely low. A large meta-analysis on endophthalmitis revealed an overall rate of 0.06% (197 of 350,535 injections) following intravitreal procedure with all anti-VEGF agents [37]. Whether the setting of administration of intravitreal therapy influences the incidence of endophthalmitis has been widely debated. IVIs with anti-VEGF agents are mainly performed in ambulatory surgery room/clean room or operating room in Europe and in other countries, such as China and India. However, a growing number of ophthalmologists in high-income countries, such as the United States, Japan, Australia, and Canada, began to perform office-based IVIs as it is a more cost-effective, convenient, and efficient approach [38–40].

The recent literature reports that endophthalmitis following anti-VEGF IVIs is low regardless of the setting where the procedure is performed. A retrospective study of 5 US retina practices including over 500,000 IVIs (bevacizumab, ranibizumab, aflibercept), all performed in an office-based setting, reported 183 cases of endophthalmitis at a rate of 0.036% [41]. An evaluation of the prospective comparison of age-related macular degeneration treatment trials (CATT) data based on 18,509 injections performed in offices showed endophthalmitis rates of approximately 0.06% [42]. Two additional retrospective studies of 10,254 and 14,895 office-based IVIs found similar endophthalmitis rates of 0.029 and 0.057%, respectively [43, 44]. A retrospective analysis including 40,011 IVIs performed in the operating room reported an endophthalmitis rate of 0.007% [45]. Freiberg et al. [46] have reported series of 134,701 IVIs performed in an operating room with laminar airflow, with a very low rate of endophthalmitis of 0.0074% per injection. A large French study (316,576 injections in a dedicated injection room) reported a 0.021% infection rate, with no significant difference observed between injections with or without filtration airflow [47]. Smaller European (40,000 injections) and German (20,179 injections) operating room-based series reported rates of 0% [48], 0.0075% [45], and 0.03% [49], with the latter cohort indicating a potential learning curve effect at 0.009% [50]. In a consecutive case series (11,710 IVIs) comparing office-based (8,647) and operating room (3,063) settings, no significant difference in endophthalmitis rates (0.035% and 0.065%, respectively) was found [51]. A meta-analysis of 25 studies investigating IVI safety with anti-VEGF agents in both settings confirmed this, reporting rates of 0.03% (95% CI, 0.03–0.04) and 0.02% (95% CI, 0.01–0.04), respectively, with no significant difference observed [39].

#### Perioperative safety

Despite extensive focus on the overall safety of intravitreal anti-VEGF injections, potential systemic complications during treatment in medical facilities, such as iatrogenic intraocular damage and complications related to the injection or procedure-induced stress, have received limited attention.

A meta-analysis assessing IVI safety with anti-VEGF agents in office-based and operating room settings revealed that ocular perioperative complications, including posterior vitreous detachment, iatrogenic/traumatic cataract, retinal detachment, and retinal tears, mainly resulted from improper injection techniques. The low occurrence rates (0%-0.67%) of these complications were independent of the setting [39]. Furthermore, the injection setting did not influence systemic safety, with no severe systemic perioperative complications reported in a study analysis totaling 1,275,815 injections [39].

Perioperative blood pressure increase has been investigated in 16 studies. All reported significant increases in systolic and/or diastolic blood pressure following intravitreal anti-VEGF therapy. Increases ranged from 5 to 18 mmHg and 3 to 7 mmHg, respectively. The incidence of systolic blood pressure rise of ⩾10 and ⩾20 mmHg was between 72%-83.8% and 46–69.5%, respectively [50, 52–60]. One study suggested the increase occurs mostly before the injection due to anxiety or alertness [60].

### Survey

Twenty centers across 12 countries on four continents reported 96,624 anti-VEGF injections in 2019. The mean number of procedures per center was 4,831 (± 1,132) (range: 607–14,980). Procedures were performed in hospital-based operating theatres (35%), dedicated clean rooms (40%), ambulatory surgery rooms (15%), and medical offices (10%). Certified ophthalmologists conducted 65% of cases, residents 30%, and trained nurses 5%. No severe perioperative systemic adverse events required critical care or emergency intervention. A cerebrovascular event (0.001%) occurred 7 days post-injection. Ocular perioperative adverse events (0.02%) included iatrogenic cataract (10), ocular hypotony (2), intraocular pressure elevation (8), and intraocular bleeding (3). Endophthalmitis cases (19, 0.02%) were reported (0.017% operating theatre, 0.019% ambulatory surgery room/clean room, 0.06% medical office).

## Discussion

This study investigated the safety of anti-VEGF IVIs and the impact of procedure settings on complications by examining national regulations and guidelines from 23 countries, reviewing relevant literature, and analyzing an international survey. Findings revealed that IVIs are predominantly administered in outpatient clean rooms (96%) or offices (39%), while Italy requires hospital-based ambulatory surgery rooms or operating theaters (4%). The risk of endophthalmitis and preoperative systemic adverse events was consistently low, irrespective of the setting, suggesting that the injection setting does not significantly affect ocular and systemic safety.

Intravitreal anti-VEGF therapy is a successful strategy for treating various retinal diseases, potentially reducing global blindness and visual impairment. Retina specialists' experience over the past decade has improved treatment effectiveness and reduced visual loss [9]. The rapid adoption of anti-VEGF agents has increased pressure on ophthalmology clinics, and with rising demand, healthcare systems must expand capacity to deliver timely, sight-saving treatments [61, 62].

A major obstacle to the full exploitation of anti-VEGF therapy is the setting in which it must be administered. The predominant setting for IVI varies between countries due to several factors, such as regulations, reimbursement policies, and traditions, which have an impact on access to care. These regulations result in IVI being performed in outpatient clean rooms (or even in the office) in some countries, while in other countries its administration is limited to ambulatory surgery rooms or operating theatres located within a hospital. The former approach is cost-effective, convenient, and efficient, and prioritizes capacity, reduction of costs, and social health, while the latter is a more conservative strategy that theoretically emphasizes patient safety but is not justified by data. Previously published evidence, based on several large-scale studies, together with the results of the present survey, showed that endophthalmitis and perioperative complications are very rare events, whose rate of occurrence does not significantly differ whether the injection is performed in the operating room, in an ambulatory surgery room, or in the office. We reported no cases of perioperative severe systemic adverse events (out of approximately 100,000 injections) and extremely low rates of perioperative ocular adverse events (0.02%) and endophthalmitis (0.02%).

A unique scenario concerns Italy. Obsolete regulations force the use of intravitreal anti-VEGF agents in hospital settings (or analogous healthcare facilities, as mentioned by the prescript). Oppositely, the international community has come to a consensus that intravitreal agents can be administered safely, comfortably, and in a more cost-efficient way in an outpatient setting, rather than in a hospital [13, 17, 24]. In Italy, defining what analogous healthcare facilities are by specifying characteristics of outpatient settings would help to implement access to intravitreal therapies.

The World Health Organization (WHO) has defined non-adherence as a major potential threat in the care of chronic diseases. Among modifiable risk factors for non-adherence, the “health system” dimension plays a major role [61]. Since anti-VEGF therapy was introduced, some countries have appropriately reorganized their national healthcare systems to improve clinic efficiencies, investing in technologies, adequate spaces, staffing, staff skills, and management planning, with adequate funding. In those countries, intravitreal pharmacotherapy has become a real game-changer, decreasing disabilities due to visual loss [9]. In other countries, strategies for optimization have not yet been implemented, rendering anti-VEGF therapy a much less effective tool.

The present study has some limitations that should be acknowledged. Due to the nature of online surveys, there is a limited ability to control and verify responses, and there is a risk of various forms of bias, such as desirability bias and response bias. Furthermore, the results of the survey may be subject to sampling error, as the sample may not be representative of the whole population of ophthalmologist/retinal specialists and may not capture the full range of opinions and experiences. Additionally, the present survey did not take into account other factors such as antibiotics prophylaxis, surgical protocols, the use of speculum, sterile drape, glove use and mask wearing, all of which can have an impact on the rate of endophthalmitis.

In conclusion, data on perioperative ocular and systemic complications do not significantly differ among various settings such as operating theatre, ambulatory surgery room, office, hospital or extra-hospital. Optimising patient management improves quality, effectiveness, and productivity, and thus increases capacity [62]. This action includes the choice of clinical spaces such as those where intravitreal therapies are administered. A successful impact of intravitreal therapy on social health strongly depends on the involvement of multiple stakeholders in the process, as well as the enhancement of existing healthcare systems and facilities, the reassessment of necessary human and technological resources, and the implementation of straightforward and up-to-date regulations which, in the end, would significantly facilitate ease to obtain care. Limited access to treatment implies undertreatment, loss of adherence, and ultimately poor results. With a strong commitment and concerted effort from healthcare professionals and policy-makers, a further reduction in new cases of blindness seems to be within reach.

### Supplementary Information

Below is the link to the electronic supplementary material.

Supplementary file1 (PDF 87 KB)

## Data Availability

Not applicable.
