# Supplementary material for: The ideal intravitreal injection setting: office, ambulatory surgery room or operating theatre? A narrative review and international survey
Source: Graefes Arch Clin Exp Ophthalmol. 2023 May 18;261(11):3299–306. doi: 10.1007/s00417-023-06108-y (PMC10587024; doi:10.1007/s00417-023-06108-y)
Supplement: Supplementary file 1 — Supplementary file1 (PDF 87 KB) [file 417_2023_6108_MOESM1_ESM.pdf]

Supplementary Table 1: Definition of settings.
Since precise definitions of different settings may vary among countries, for the purpose of this survey, the most widely-used definitions are being used.

Category	Setting	Definition
Category 1	Operating theatre	A specific area in hospitals or other healthcare facilities where surgical operations are performed in an aseptic environment. It must meet certain standards which include requirements for ventilation, lighting, cleanliness, and infection control.
Category 2	Ambulatory Surgery Room	Area designed for the provision of surgical procedures that do not require hospitalisation. It is typically located within a clinic or hospital and contains the necessary equipment and personnel to perform minor surgeries in a sterile environment.
	Clean Room	A dedicated room in medical facilities that is kept free from any outside contamination and is used exclusively for the preparation and administration of intravitreal injections. It is typically designed to have a low level of airborne contaminants, dust and other particles, and be free of any potential pathogenic organisms.
Category 3	Office	A facility where ophthalmologists provide diagnosis and treatment in a non-sterile environment.

Supplementary Table 2: Intravitreal Injection Setting Group (I^2SG) Survey

Question 1: In which country do you primarily work?
Question 2: According to the rules and guidelines of your country, in what setting should intravitreal anti-VEGFs be administered?
Question 3: Are there any limitations or specific rules for the use of anti-VEGF drugs (both in-label and off-label) in your country?
Question 4: In what setting do you (or your team) perform the majority of intravitreal anti-VEGF injections?
Question 5: Which type of personnel performs the majority of intravitreal injections in your institution?
Question 6: How many injections did you and your team perform in 2019?
Question 7: How many cases of severe perioperative systemic adverse events have you recorded in 2019 following an intravitreal injection?
Question 8: If you recorded at least one case of severe perioperative adverse event, please specify the type and the outcome.
Question 9: How many cases of endophthalmitis following an intravitreal injection did you record in 2019?
Question 10: How many cases of ocular perioperative adverse events were recorded in 2019?
Question 11: If at least one case of ocular perioperative adverse event was recorded, please specify the type and outcome.

Legend: VEGF: vascular endothelial growth factor
